# Increased Thrombotic Risk in COVID-19: Evidence and Controversy

**DOI:** 10.3390/jcm12134441

**Published:** 2023-07-01

**Authors:** Antonio De Vita, Francesco Franceschi, Marcello Covino

**Affiliations:** 1 Department of Cardiovascular Sciences, Fondazione Policlinico Universitario A. Gemelli IRCCS, Università Cattolica del Sacro Cuore, 00168 Roma, Italy; 2 Department of Emergency Medicine, Fondazione Policlinico Universitario A. Gemelli IRCCS, Università Cattolica del Sacro Cuore, 00168 Roma, Italy; francesco.franceschi@unicatt.it (F.F.); macovino@gmail.com (M.C.)

## 1. Introduction

The pandemic of respiratory disease caused by the novel coronavirus named SARS-CoV-2, which emerged at the end of 2019, is still ongoing [1,2]. Many scientific reports worldwide have contributed to the comprehensive understanding of the disease and its related complication, which significantly affect the prognosis [3,4]. It is widely known that although pulmonary involvement is the most typical feature of COVID-19, severe infection may result in a prothrombotic state attributable to excessive inflammation, cytokine storm, hypoxia, and prolonged immobilization, predisposing patients to both venous and arterial thromboembolic events, with an adverse impact on in-hospital prognosis [5,6,7,8].

As a result of personal and institutional precautions, along with the ongoing vaccination campaign, there has been a decrease in the number of patients with severe respiratory disease caused by COVID-19, as well as a reduction in morbidity, mortality, and the need for hospitalization. During the second and third waves of the disease, the implementation of “grey areas” within Emergency Departments enabled the identification of distinct subgroups of infected patients. This has helped physicians differentiate patients who are at a high risk of developing severe lung failure and thrombotic complications [9,10].

The latest clinical data indicate that, in COVID-19 patients, pulmonary embolism (PE) and deep vein thrombosis (DVT) are the most frequent thrombotic events [11]. Interestingly, hospitalized patients still report a significant risk of venous thromboembolism (VTE) despite receiving anticoagulation prophylaxis (with rates as high as 25–30% in critically ill and mechanically ventilated patients) [12,13]. Additionally, other thrombotic complications, such as stroke, acute limb ischemia, and acute coronary syndrome (ACS), have been reported in approximately 5–20% of patients admitted to the hospital [14].

## 2. Potential Mechanisms of COVID-19-Induced Thrombosis

The close link between COVID-19 and vascular coagulopathy suggests that there may be several dysregulated molecular pathways contributing to the development of thrombosis during the progression of the disease. Notably, the downregulation of ACE2 leading to RAAS augmentation represents a significant factor [15]. Additionally, a dysregulated immune and inflammatory response is a well-established risk factor for blood coagulation [16]. It is plausible that both factors synergistically amplify the risk of thrombosis in COVID-19 patients, resulting in a “double whammy” effect [17].

In particular, when human cells are internalized by SARS-CoV-2, ACE-2 activity is likely to be diminished, resulting in elevated levels of angiotensin II and reduced levels of angiotensin 1–7 [18]. It is important to remember that while angiotensin II has pro-inflammatory and pro-thrombotic effects, angiotensin 1–7 is now recognized as a crucial anti-inflammatory and anti-thrombotic peptide by stimulating the production of nitric oxide and prostacyclin, which inhibits platelet activation [18]. Furthermore, ACE-2 is known to be widely expressed on endothelial cells, indicating the possibility of direct infection of endothelial cells by SARS-CoV-2 [19].

From another perspective, several studies have highlighted alterations in both innate and adaptive immunity among COVID-19 patients, accompanied by a significant release of pro-inflammatory cytokines, commonly referred to as a “cytokine storm”. This dysregulated innate immune response subsequently activates various pathways, known as “immune-thrombosis”, which can potentially lead to an increased risk of thrombosis [20,21,22].

## 3. Clinical Aspects of Coagulopathy Associated with COVID-19

Despite anticoagulant administration, recent studies reported high rates of VTE, ranging from 25% to 70% in critically ill patients in the intensive care unit (ICU), whereas the PE incidence varies from 5% to 25% [12,13]. Autopsy studies have shown bilateral DVT in a significant number of COVID-19 deaths, with some cases demonstrating massive PE as the cause of death [23]. Asymptomatic DVT has been found in up to 85% of critically ill patients screened with ultrasound [24]. A meta-analysis of 86 studies reported a VTE prevalence of 7.9% in non-ICU patients and 22.7% in ICU patients, respectively, with a PE prevalence ranging from 3.5% to 13.7% [25]. However, there are currently no data on the incidence of VTE in outpatients with less severe COVID-19 disease, and the evidence regarding arterial thromboembolism in COVID-19 is still evolving. Additionally, there have been reports of thrombotic microangiopathy, indicating that microvascular thrombotic injury could be responsible for renal and neurological dysfunction associated with COVID-19 [26]. Rare instances of thrombotic events accompanied by thrombocytopenia, known as vaccine-induced immune thrombotic thrombocytopenia (VITT) or thrombosis with thrombocytopenia syndrome (TTS), were observed as a complication after administration of the recombinant adenoviral vector vaccine (ChAdOx1 nCov-19, AstraZeneca), with a significantly higher rate of uncommon thrombosis, particularly involving cerebral and splanchnic veins [27]. The administration of mRNA vaccines (Comirnaty/BioNTech/Pfizer and mRNA-1273/Moderna) has been associated with a significantly lower rate of thrombotic complications [28]. The pathophysiological basis of VITT/TTS may concern the activation of macrophages by the spike protein of COVID-19 vaccines, with consequent inflammation, release of platelet factor 4 (PF4) from platelets and downregulation of ACE2 on the endothelial surface [29].

Several governmental and professional associations have issued guidelines regarding the screening, prevention, and treatment of VTE in hospitalized patients with COVID-19 [30,31,32,33]. Typically, patients admitted for medical reasons would undergo risk stratification using assessment models such as PADUA or IMPROVE, and those deemed to be at higher risk for VTE and low risk for bleeding would receive prophylaxis with subcutaneous unfractionated heparin (UFH) or low-molecular-weight heparin (LMWH) at standard doses, as outlined in the published guidelines [30,31,32,33]. Given the increased risk of VTE observed in hospitalized COVID-19 patients according to observational studies, most guidelines recommend anticoagulant prophylaxis for VTE in all patients without a high risk of bleeding [30,31,32,33]. It is not recommended to routinely screen for lower-extremity VTE using Doppler ultrasound. Nonetheless, clinicians should be cautious and consider ordering appropriate diagnostic tests and starting therapeutic anticoagulation if PE is suspected, particularly in case of worsening oxygenation and a rapid increase in D-Dimer levels.

## 4. Pharmacologic Rationale and Evidence for Thromboprophylaxis in COVID-19 Patients

Heparins, aside from their anticoagulant properties, have a well-established history of anti-inflammatory activity. Previous studies have shown that heparins can reduce levels of IL-6 and IL-8, as well as mitigate damage to human pulmonary microvascular endothelial cells associated with sepsis [34]. Heparin also inhibits neutrophil chemotaxis and eosinophil migration and prevents the adhesion of leukocytes to endothelium, which is a crucial early step in sepsis [35]. Furthermore, emerging evidence suggests that heparin plays a role in reducing the infectivity of SARS-CoV-2. As a sulfated polysaccharide, heparin exhibits a high binding affinity to the spike protein of SARS-CoV-2 in vitro. This suggests that heparins could act as decoys, preventing the virus from binding to heparan sulfate co-receptors in host tissues. Recent findings indicate that both UFH and LMWH bind to and destabilize the receptor binding domain of the SARS-CoV-2 spike protein, directly inhibiting the binding of the spike protein to the ACE2 receptor at therapeutic concentrations [36].

Heparins have demonstrated efficacy in both preventing and treating VTE in hospitalized patients with acute illnesses. Given the heightened risk of thrombotic complications in COVID-19 and the unique pharmacological properties of heparins, numerous studies have been performed to explore additional clinical outcomes such as reduced hospitalizations, progression to mechanical ventilation and mortality. Several retrospective studies have described a potential survival advantage associated with both UFH and LMWH at both prophylactic and therapeutic doses, particularly in patients with severe COVID-19 indicated by elevated D-dimer levels or the need for mechanical ventilation [37,38,39].

The results of heparin administration have also been extensively investigated through numerous randomized trials [40]. In the HESACOVID Trial, Lemos et al. demonstrated, in a small cohort of severely ill patients, a significant reduction in the need for mechanical ventilation and improved blood gas parameters in patients treated with 14-days of therapeutic anticoagulant dosage (LMWH or UFH) compared to patients receiving thromboprophylaxis dosage [41]. Conversely, in the INSPIRATION trial, the authors failed to show significant differences in the primary composite outcome of venous or arterial thrombosis, treatment with extracorporeal membrane oxygenation (ECMO), or all-cause mortality, between patients treated with enoxaparin at an intermediate dose (1 mg/kg/daily) and patients receiving only prophylactic dosage (enoxaparin, 40 mg daily) [42]. Likewise, the multicenter randomized AntiCoagulaTlon cOroNavirus (ACTION) trial failed to show a significant benefit of a therapeutic dose of anticoagulation compared to prophylactic dose in terms of mortality, length of hospitalization, or duration of oxygen therapy after 30 days, with a significant increase observed in major or clinically relevant non-major bleeding [43]. Similar findings were reported in other randomized clinical trials and meta-analyses [44,45,46,47,48].

The efficacy and safety of other anticoagulants in improving COVID-19 outcomes have been examined. In two studies, the prophylactic dose of fondaparinux (2.5 mg daily) demonstrated similar effectiveness and safety compared to prophylactic enoxaparin dosage [49,50]. However, another study found a significantly higher rate of major or clinically relevant bleeding in patients treated with fondaparinux [51].

Furthermore, a recent meta-analysis comparing 12 studies and a total of 30,646 patients found that clinical outcomes were not improved by pre-existing direct oral anticoagulants (DOAC) therapy at the time of COVID-19 diagnosis [52].

## 5. Prophylaxis and Treatment of Thrombotic Complications in COVID-19 Patients: Current Practice Recommendation

When selecting a preventive medication, some guidelines suggest prioritizing LMWH over UFH for practical reasons such as minimizing the frequency of injections and reducing the potential exposure of medical professionals. However, in cases of severe chronic kidney disease (CKD), UFH is favored over LMWH. The use of DOACs for VTE prophylaxis is not recommended due to potential drug interactions and longer half-lives, which may complicate hemostasis management after surgery or invasive procedures. Most guidelines advise standard anticoagulant prophylaxis doses for non-critically ill hospitalized patients, whereas intermediate doses are suggested for obese and critically ill patients [30,31,32,33]. In patients with suspected heparin-induced thrombocytopenia, fondaparinux may represent a valuable alternative, whereas mechanical prophylaxis with intermittent pneumatic compression (IPC) is advised for patients in whom the use of anticoagulants is absolutely contraindicated [30,31,32,33]. The Global COVID-19 Thrombosis Collaborative Group is the only guideline that addresses non-hospitalized patients with COVID-19, recommending increased mobility and considering anticoagulant prophylaxis for those with limited mobility, a history of VTE, or active malignancy [31]. Routine post-hospital discharge prophylaxis is not recommended by the National Institutes of Health (NIH) or CHEST guidelines [30,31,32,33]. In limited cases, guidelines recommend the use of either enoxaparin or rivaroxaban for 14–45 days after hospital discharge.

Hospitalized patients who have received a confirmed diagnosis of DVT or PE are eligible to receive initial parenteral anticoagulation treatment, followed by a transition to DOACs [27,28,29,30]. When using therapeutic doses of UFH, monitoring of anti-Xa levels instead of aPTT is recommended by most guidelines, as factor VIII and elevated levels of positive lupus anticoagulants, with consequently prolonged aPTT, are very common.

Lastly, the treatment of VITT includes anticoagulation, a high dose of intravenous immunoglobulin, therapeutic plasma exchange, corticosteroids, rituximab and eculizumab. Current guidelines recommend avoiding heparin-based anticoagulation, due to the presence of anti-PF4 antibodies, and preferring non-heparin anticoagulants such as fondaparinux, DOACs or direct thrombin inhibitors (e.g., argatroban, bivalirudin). However, recent studies have suggested that the use of heparin may be safe in managing VITT [53,54,55].

## 6. Conclusions

In conclusion, due to a lack of data regarding the safety of therapeutic versus prophylactic anticoagulation and the existence of several guidelines, an individualized approach is advised, considering the balance between benefits and bleeding risk. Indeed, while recent trials have shown notable progress, clinicians need to take into account the limitations that restrict their generalizability and the various manifestations of this disease. Although therapeutic anticoagulation has not definitively demonstrated mortality benefits, it does notably decrease the occurrence of VTE in COVID-19 patients. However, the optimal timing for initiating anticoagulation during disease progression is still unclear. Studies investigating the role of anticoagulation across all stages of COVID-19 patient care, including ambulatory, hospitalized, and post-hospital settings, are currently underway. Hopefully, they will provide valuable insights and address the remaining uncertainties.
